# The Importance of a Complete Differential: Case Report of a Tuberculoma in a Patient without Pulmonary Involvement

**DOI:** 10.7759/cureus.1405

**Published:** 2017-06-28

**Authors:** Pooja Sethi, Jennifer Treece, Chidinma Onweni, Vandana Pai, Zia Rahman, Siddharth Singh

**Affiliations:** 1 Cardiology, Quillen College of Medicine, East Tennessee State University; 2 Internal Medicine, Quillen College of Medicine, East Tennessee State University; 3 Cardiology, Cedar Sinai Medical Center

**Keywords:** tuberculoma, brain mass, ring-enhancing lesion, headache

## Abstract

Patients with a tuberculoma typically present with pulmonary involvement of tuberculosis and have risk factors for tuberculosis (TB). The risk factors for tuberculosis include bacillary load, proximity to infectious case, immunosuppressive conditions, malnutrition, young age, diabetes mellitus, working in healthcare, recent incarceration, alcohol use, and tobacco use. Although rare, it is possible for a patient to present with a tuberculoma despite the absence of risk factors for tuberculosis other than diabetes and without pulmonary involvement.

## Introduction

Tuberculosis (TB) is a bacterial infection caused by *Mycobacterium tuberculosis* [1-2]. It is most often an infection of the lungs but can infect other organs in the body, and it is spread by droplets from the respiratory tract of a patient with active tuberculosis [3]. Extrapulmonary TB (EPTB) refers to disease involving organs other than the lungs: pleura, lymph nodes, abdomen, genitourinary tract, skin, joints, bones, skeletal, or meninges. The spinal column is involved in less than one percent of all cases of TB. Spinal TB, also called Pott's disease, is the most common as well as one of the most dangerous forms of skeletal TB. It is reported to account for 50% of all cases of skeletal TB. Approximately 1% of patients infected with *Mycobacterium tuberculosis* develop central nervous system (CNS) involvement, which may manifest as a tuberculoma, meningitis, or as an abscess [4]. Of the extrapulmonary manifestations of tuberculosis, CNS involvement is the most severe and has a high rate of mortality as well as neurologic residual sequelae despite treatment [5]. For this reason, when evaluating common complaints such as headache, a broad differential diagnosis should be considered. Tuberculoma should be included in the differential diagnosis of an intracranial mass even if the patient does not possess risk factors for tuberculosis and does not have any pulmonary involvement.

## Case presentation

**Figure 1:**
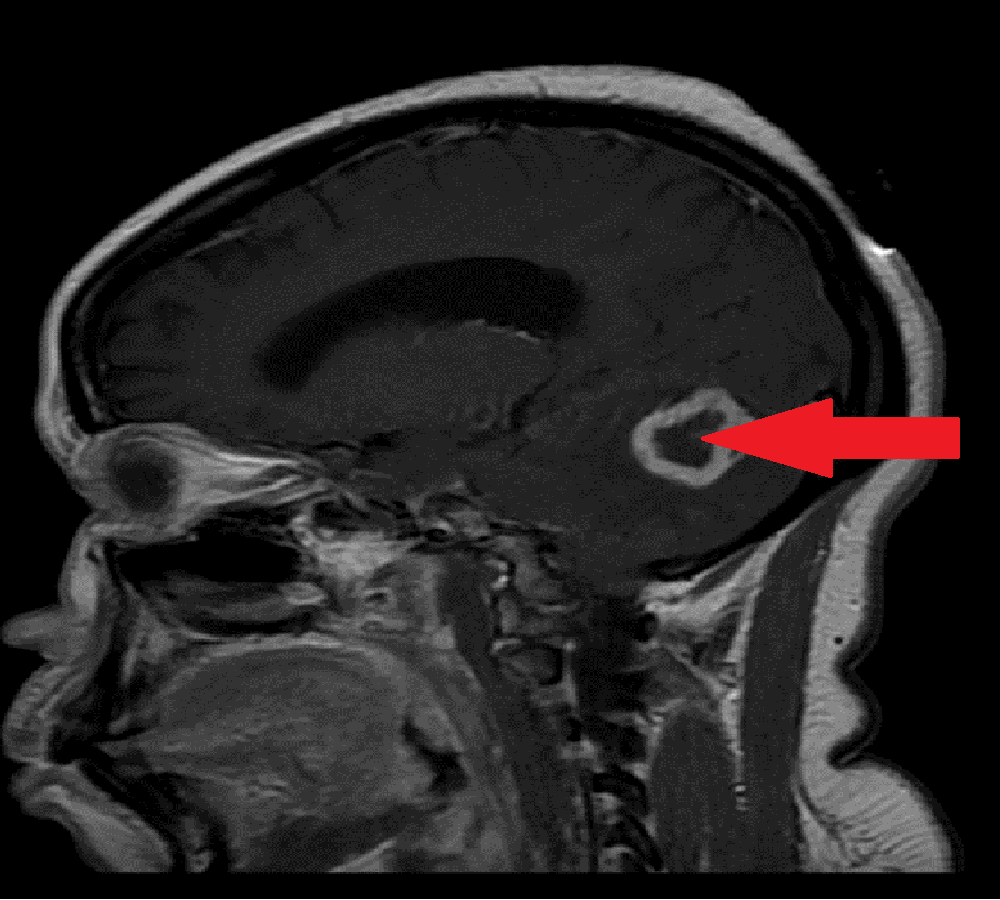
MRI of the brain (sagittal view) shows a large right cerebellar ring-enhancing lesion indicated by the arrow. MRI of the brain from the side (sagittal view) shows a large ring-enhancing mass in the cerebellum, which is seen as an irregular oval-appearing lesion with a bright white outline and is indicated by the arrow. MRI - magnetic resonance imaging.

**Figure 2:**
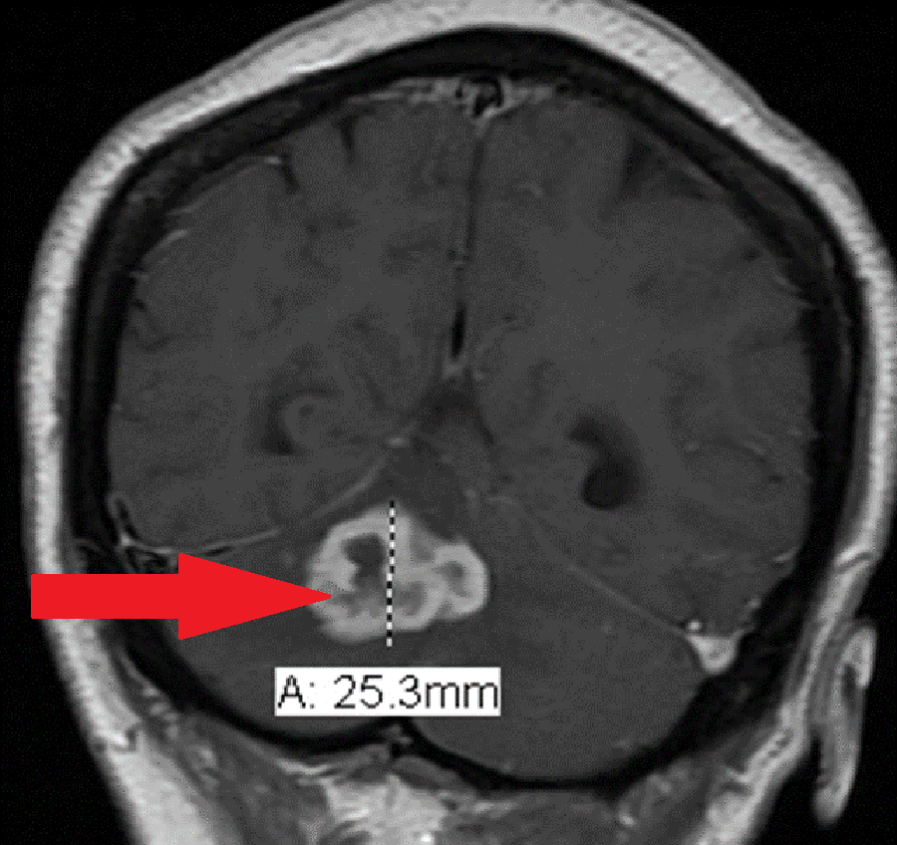
MRI of the brain (coronal view) shows a right cerebellar ring-enhancing lesion. MRI of the brain from a vertical, frontal plan (coronal view) showing a large (25.3 mm diameter) ring-enhancing lesion in the patient's cerebellum, which is indicated by the arrow. MRI - magnetic resonance imaging.

**Figure 3:**
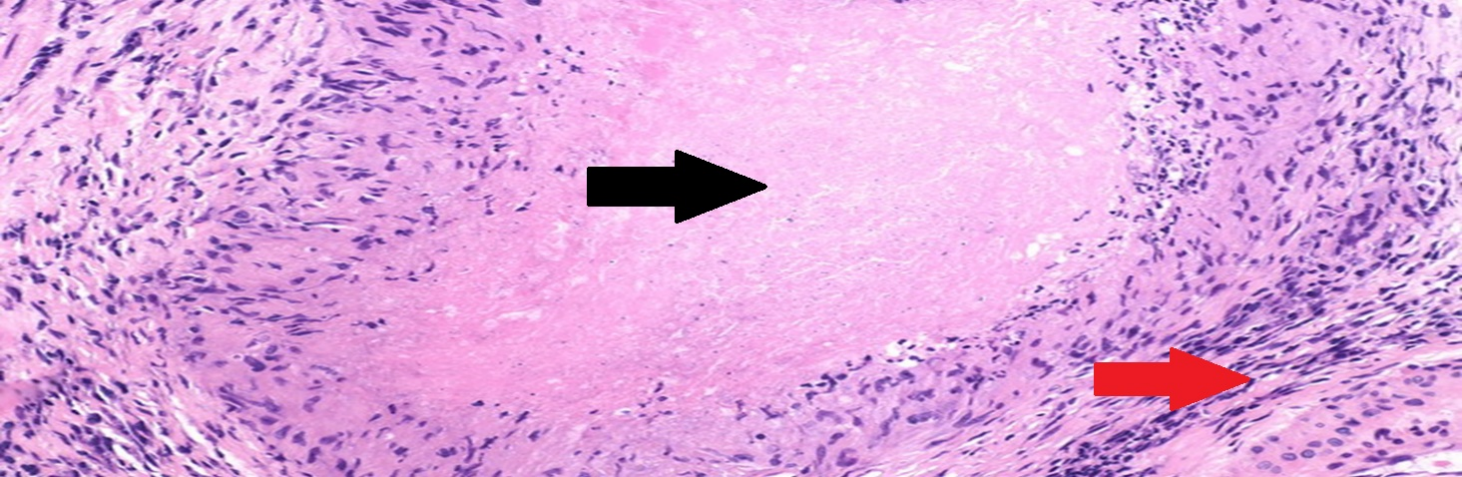
Necrotizing granulomas on histopathology Dissection of the large ring-enhancing lesion removed from the patient's cerebellum showed necrotizing granuloma when the tissue was viewed under the microscope. The pink central area is necrosis, indicated by the black arrow, with surrounding epithelioid histocytes, indicated by the red arrow. Necrotizing granuloma is found in tuberculosis.

A 48-year-old African American female presented with a one-month history of headaches and nausea. Her only significant past medical history was diabetes. Her social history was negative for such risk factors as a high-risk profession in the healthcare field, recent incarceration, known contact with persons diagnosed with active tuberculosis or recent travel to areas in which tuberculosis is endemic. Her physical exam was unremarkable, and she had no focal neurological deficits. She was afebrile and hemodynamically stable. She presented to an outside facility where she had a computerized tomography (CT) scan of her head performed, which showed a posterior fossa mass with moderate hydrocephalus. This was followed by a magnetic resonance imaging (MRI) of the brain with and without contrast, which revealed a large right cerebellar ring-enhancing mass (Figures 1-2). The primary concern at that time was that this mass could possibly be a primary brain glioma like a high-grade astrocytoma or a medulloblastoma. The patient was transferred to another hospital for neurosurgical evaluation and excision of the mass. She underwent an exploratory posterior fossa craniectomy, cerebellar exploration and decompression, but no mass was identified at that time. Due to persistent headaches, she then had a repeat exploration of posterior fossa 15 days later, and identification and excision of the cerebellar mass occurred. Histopathology from the procedure revealed necrotizing granulomas (Figure 3), and stains specific for acid fast bacilli and fungus were both negative. Subsequently, the brain tissue grew *Mycobacterium tuberculosis*. The patient reported no prior knowledge of having tuberculosis, nor did she endorse exposure to patients with active tuberculosis. She previously had purified protein derivative (PPD) tests for employment, which had been negative. She was not immunocompromised and had recently been tested negative for human immunodeficiency virus (HIV) six months prior.

## Discussion

Epidemiology

Approximately 10-15% of all cases of tuberculosis involve extrapulmonary tuberculosis without lung involvement. CNS tuberculosis accounts for 1% of all tuberculosis cases. Tuberculoma account for 0.2% of all biopsied brain masses [3].

Risk factors/associations

Pulmonary tuberculosis accounts for approximately 40-60% of tuberculoma cases. Patients with human immunodeficiency virus (HIV) have five times greater risk of tuberculoma than non-HIV patients [1]. Immunodeficiency is a risk factor for the development of tuberculosis as is organ transplantation due to chronic use of immunosuppressant medications. Recipients of a solid organ transplantation are 20-74% more likely to develop tuberculosis [6]. Eight percent of cases of tuberculoma are associated with the patient having concurrent diabetes, which was the sole risk factor for the patient in this case [1].

Clinical features

When a tuberculoma is large in size, it can mimic a brain tumor compressing surrounding brain tissue and causing symptoms of increased intracranial pressure (ICP), such as headache, nausea, and vomiting, which were all present in the patient presented in this case. Other possible symptoms that can occur with a tuberculoma but were not present in the patient in this case include seizures and cranial nerve palsies. These are common presentations of tuberculomas due to irritation and compression of surrounding brain parenchyma [7]. Tuberculomas that are large enough to cause compression symptoms are rare [8].

Diagnosis

Diagnostic imaging with a CT and MRI of the brain show ring-enhancing lesions with intense surrounding edema. Although multiple lesions may be present in up to one-third of patients with a tuberculoma, the patient presented in this case had a solitary brain lesion [9].

The differential diagnosis for an intracranial tuberculoma includes primary brain malignancy (high-grade astrocytoma or medulloblastoma), metastatic lesion, sarcoidosis, toxoplasma, granulomatous diseases, and fungal infections [8].

The gold standard for diagnosis of a tuberculoma is a brain mass biopsy with histopathologic evaluation [8]. Necrotizing granulomas are often found on histology. The biopsy should be assessed for acid fast bacilli (AFB), though it was negative in the patient presented. The tissue biopsy should also be cultured, which will result in the growth of *Mycobacterium tuberculosis* [1].

Differential diagnosis

High-grade astrocytoma or a medulloblastoma are on the list of differential diagnoses.

Treatment

Surgical resection of the mass with decompression is desired when the patient has signs and symptoms of increased intracranial pressure, which occurred in the patient presented in this case. Following surgical resection, the patient will need anti-tubercular treatment for 18 months with rifampicin, isoniazid, pyrazinamide, and ethambutol medication cocktail [2]. Clinical recovery is expected to begin within weeks of medication initiation, but it will take between six to twelve months for radiographic evidence of the mass to reduce in size [9]. It is possible for a new intracranial lesion to develop or the existing lesion to expand while the patient is on anti-tubercular medications. This occurs through a suspected paradoxical immune-mediated response. These patients need to have systemic steroids added to their treatment regimen for four to eight weeks in addition to the anti-tubercular medications, which do not need to be changed as this does not indicate failure in treatment [10].

## Conclusions

Extrapulmonary tuberculosis without lung involvement is rare and accounts for 10-15% of all tuberculosis cases. CNS tuberculosis accounts for only 1% of all tuberculosis cases. The patient presented in this case did not have predisposing factors for a tuberculoma given that her HIV test and PPD were negative. Her age was also atypical for tuberculomas as they are usually seen in children. She was diabetic, which is associated with 8% of all reported cases of tuberculomas. Biopsy and histopathology is the gold standard in diagnosing tuberculomas as imaging studies can often be misleading in the diagnosis of this rare condition. Lumbar puncture is not usually possible given the raised intracranial pressure.

Final learning objectives

CNS involvement of tuberculosis is difficult to diagnose and treat and has elevated rates of morbidity and mortality associated with it if not diagnosed and treated adequately early in the disease course as patients who present late in the disease course have poorer outcomes.

CNS tuberculoma needs to be considered in patients who present with a brain mass even if they are not known to have tuberculosis and have minimal risk factors for tuberculosis.
